# The Oxidative Status and Na^+^/K^+^-ATPase Activity in Obsessive-Compulsive Disorder: A Case Control Study

**DOI:** 10.1155/2024/9979582

**Published:** 2024-02-23

**Authors:** Amir Hossein Mohammadi, Ebrahim Balandeh, Jila Hasani, Mohammad Karimian, Vajiheh Arabshahi, Morteza Pourfarzam, Fereshteh Bahmani, Gholamreza Namazi

**Affiliations:** ^1^Research Center for Biochemistry and Nutrition in Metabolic Diseases, Institute for Basic Sciences, Kashan University of Medical Sciences, Kashan, Iran; ^2^Student Research Committee, Kashan University of Medical Sciences, Kashan, Iran; ^3^Neuroscience Research Center, Institute of Neuropharmacology, Kerman University of Medical Sciences, Kerman, Iran; ^4^Department of Molecular and Cell Biology, Faculty of Basic Sciences, University of Mazandaran, Babolsar, Iran; ^5^Department of Nutrition, School of Public Health, Iran University of Medical Sciences, Tehran, Iran; ^6^Department of Clinical Biochemistry, School of Pharmacy and Pharmaceutical Sciences, Isfahan University of Medical Sciences, Isfahan, Iran; ^7^Department of Clinical Biochemistry, School of Medicine, Kashan University of Medical Sciences, Kashan, Iran

## Abstract

**Background:**

Oxidative stress is involved in pathogenesis of some psychiatric disorders. To examine the role of oxidative stress in the etiopathogenesis of obsessive-compulsive disorder (OCD), we aimed to determine oxidative stress indices, including malondialdehyde (MDA) levels in serum and red blood cells (RBC) membrane, total antioxidant capacity (TAC), serum glutathione (GSH) levels, serum antioxidant vitamins (A and E), and Na^+^/K^+^-ATPase activity, in patients with the mentioned disorder vs. healthy controls.

**Method:**

39 OCD patients diagnosed based on Diagnostic and Statistical Manual of Mental Disorders (DSM-V) and 39 volunteer healthy subjects were included in this study. MDA levels in serum and RBC membrane were measured using fluorometric method. Serum TAC level, serum GSH level, and Na^+^/K^+^-ATPase activity were also measured using spectrophotometric methods. Serum levels of vitamins were calculated by reversed-phase high-performance liquid chromatography (RP-HPLC).

**Result:**

There was a significantly higher MDA level in serum (*p* < 0.0001) and RBC membrane (*p* = 0.002) of OCD patients compared with those in controls. A significant reduction in vitamin A (*p* = 0.001) and vitamin E (*p* = 0.024) levels was found in OCD patients vs. controls. There was significantly lower activity of erythrocyte membrane Na^+^/K^+^-ATPase in RBC membrane of OCD patients vs. controls (*p* < 0.0001).

**Conclusion:**

Our findings indicate significantly higher levels MDA in both serum and RBC membrane, lower levels of serum vitamins A and E, and lower activity of membrane Na^+^/K^+^-ATPase in OCD patients compared to controls. These suggest an imbalance between oxidant and antioxidant factors in OCD patients that might play a fundamental role in the etiopathogenesis of OCD.

## 1. Introduction

Obsessive-compulsive disorder (OCD) is a relatively common neuropsychiatric disorder identified by persistent thoughts and/or ritualized repetitive behaviors that are done in response to set rules or obsessions intending to reduce distress [1, 2]. The worldwide prevalence of OCD is reported to be 2–3%. It has also been considered to be among the top 10 most debilitating situations by the World Health Organization (WHO) [3–5]. OCD patients have many problems in neurotransmitter system [6, 7], immune system [8, 9], and sleep [10] and lower IQ [11] and higher suicide [12–15] than people without OCD. OCD could be considered as a phenotypically heterogeneous disorder manifested by various symptoms [16]. These symptom dimensions which overlap sometimes are captured in the Yale-Brown Obsessive-Compulsive Scale (Y-BOCS), a widely used clinical measure providing a checklist of OCD symptoms that yields a severity score for obsessive, compulsive, and total symptoms [17, 18]. The etiology of OCD remains elusive. Oxidative stress, which resulted from an imbalance between the enhanced reactive oxygen species (ROS) level and decreased antioxidant protection, exerts a fundamental role in the pathophysiology of OCD [19]. Recently, ROS are thought to be involved in the pathogenesis of several diseases, including neuropsychiatric disorders [20]. Increased ROS levels manifested during oxidative stress can lead to neuronal damage due to peroxidation of nucleic acids, lipids, and proteins that can result in the disruption of the membrane integrity and changes in the natural neuronal apoptosis [21]. For instance, enhanced lipid peroxidation could alter several features of the cell membrane such as fluidity as well as the function of some membrane enzymes (e.g., Na^+^/K^+^-ATPase activity) [22].

The Na^+^/K^+^-ATPase pump is an enzyme that exists in cellular plasma membrane which is sensitive to alterations in membrane fluidity and is responsible for management of nutrient uptake, cell volume, and hydration [23]. A previous study showed that Na^+^/K^+^-ATPase is involved in bipolar disorder [24].

Currently, there is scant data regarding Na^+^/K^+^-ATPase activity and lipid peroxidation of red blood cell (RBC) membrane in the case with OCD. Thus, we aimed to determine the levels of oxidative stress indices, including malondialdehyde (MDA) levels in serum and RBC membrane, total antioxidant capacity (TAC), serum glutathione (GSH) levels, serum antioxidant vitamins (A and E), and Na^+^/K^+^-ATPase activity, in cases with and without OCD.

## 2. Material and Method

### 2.1. Subjects

In this study, participants were 39 patients with OCD (women 28, men 11) who were recruited from psychology service clinic (Kashan, Iran) and 39 control subjects without OCD (women 33, men 6). The OCD status was diagnosed based on DSM-V criteria (according to Structured Clinical Interview for DSM-5 Disorders (SCIDs)) by an experienced clinical psychologist. Also, all participants were interviewed for other psychiatric disorders. The presence and severity of obsessive and compulsive types were investigated through the structured interview using the Yale-Brown Obsessive-Compulsive Scale (Y-BOCS) checklist and severity scale. All participants were aged between 18 and 60 years and were receiving routine treatment of OCD. Subjects were also excluded for any of the following: having symptoms for less than six months, having any other mental or physical illness, blood transfusion or surgical procedures in the preceding 3 months, use of antidepressants, alcohol consumption, smoking, and prior treatment with antioxidant compounds. Pregnant women or women who were menstruating were also excluded from the study. All participants were matched regarding gender, age, and nutritional status by N4 software. The current project was approved by the Ethical Committee of Kashan University of Medical Sciences (IR.KAUMS.MEDNT.REC.1399.057), and participants were enrolled after getting signed informed consent. All methods were performed in accordance with the relevant guidelines and regulations.

### 2.2. Product Number

The materials used in this research are as follows: 1,3,3-tetraethoxypropane (Sigma, CAS number 122-31-6), 2-thiobarbituric acid (Merck, CAS number 504-17-6), 5,5′-dithiobis(2-nitrobenzoic acid) (Sigma, CAS number 69-78-3), acetic acid (Merck, CAS number 64-19-7), acetonitrile (Merck, CAS number 75-05-8), EGTA (Sigma, CAS number 67-42-5), FeCl_3_ (Sigma, CAS number 7705-08-0), FeSO_4_ (Sigma, CAS number 7720-78-7), KCl (Merck, CAS number 7447-40-7), methanol (Merck, CAS number 67-56-1), MgCl_2_ (Merck, CAS number 7786-30-3), Na2EDTA (Merck, CAS number 6381-92-6), NaCl (Sigma, CAS number 7647-14-5), ouabain (Merck, CAS number 11018-89-6), sodium citrate (Merck, CAS number 6132-04-3), sodium dodecyl sulfate (Merck, CAS number 151-21-3), tetrahydrofuran (Merck, CAS number 109-99-9), TPTZ (Sigma, CAS number 3682-35-7), trichloroacetic acid (Merck, CAS number 76-03-9), and Tris hydrochloride (Sigma, CAS number 1185-53-1).

### 2.3. Collection and Preparation of Blood Samples

From each participant, about 10 mL of whole blood (volume 10 mL) was drawn from the basilic vein after a 12–14 h fasting. About 6 mL of taken blood was collected in heparinized tubes (10 units per milliliter of blood) in order to be used for preparation of packed erythrocyte. The rest of the taken blood was collected in tubes including colt activator in order to isolate serum. For serum preparation, about 4 mL of collected blood was centrifuged (3,000 RPM; 5 min; 4°C) and the derived serum was removed and stored at –70°C into two 0.5 mL aliquots for future biochemical analyses. Indeed, heparinized blood was centrifuged (1,500 g; 10 min) to isolate plasma and prepare packed erythrocyte. The plasma, buffy coat, and red blood cells were removed. The remained erythrocytes were resuspended and washed twice in Tris-buffered saline, that is, 20 mM Tris-HCl, pH 7.5 containing 145 mM NaCl. After centrifugation at 2,000 g for 15 min, the washed packed erythrocytes were utilized for membrane isolation [25].

### 2.4. Biochemical Analysis

Total cholesterol, triglyceride, and low-density lipoprotein- (LDL-) and high-density lipoprotein- (HDL-) cholesterol levels were measured by standard methods using commercial kits (Parsazmun Co., Tehran, Iran) and an automated analyzer (BS800, China).

### 2.5. Ferric Reducing Antioxidant Power (FRAP) Assay

Serum FRAP was measured using Benzie and Strain's method [26, 27]. The working FRAP reagent was made by mixing acetate buffer (300 mM, pH 3.6), 10 mM TPTZ (2,4,6-tri (2-pyridyl)-1,3,5-triazine) solution in 40 mM HCl, and FeCl_3_·6H_2_O solution (20 mM) in proportions of 10 : 1 : 1 (*v*/*v*). Samples (25 *μ*L) were mixed with 750 *μ*L of fresh FRAP reagent. Absorbance was measured after 5 min at 593 nm using FRAP working solution as a blank. A calibration curve was made by an aqueous solution of ferrous sulfate (FeSO_4_·7H_2_O).

### 2.6. Determination of GSH Levels

Serum GSH levels were measured using Beutler and Gelbart spectrophotometrical method [28, 29]. The assay mixture contained 100 *μ*L of sample, 0.5 mL of 5-5′-dithiobis-2-nitrobenzoic acid (40 mg in 100 mL dH_2_O containing 1% sodium citrate), and 0.5 mL of 0.3 M phosphate buffer (pH 8.0). The absorbance was measured after 5 min at 412 nm.

### 2.7. Isolation of Erythrocyte Ghost Membrane

Hypotonic lysis of packed cells was used to prepare erythrocyte ghost membrane. Around 2.5 mL of washed packed erythrocytes was diluted by 10 volumes of ice cold 5 mM Tris and Na2EDTA (0.1 mM; pH 7.6). This solution was mildly revolved and then was kept for 15 min at 4°C. In the next step, the hemolysated mixture was centrifuged (20,000 g; 20 min; 4°C) and the supernatant eliminated. The residual matter was washed 3 times with NaCl (17 mM) and Tris-HCl (5 mM; pH 7.6) and then 3 times with Tris-HCl (10 mM; pH = 7.5) and so was centrifuged at 20,000 g. In the next step, the pellet was resuspended in Tris-HCl (10 mM; pH = 7.5) [30]. Multiple aliquots (0.2 mL) of this hemoglobin-free membrane suspension were closely frozen in liquid nitrogen and kept at –70°C until evaluation of membrane protein and lipid peroxidation. Protein concentration of erythrocyte membrane was estimated using modified Lowry method described by Markwell et al. [31].

### 2.8. Measurement of Erythrocyte Membrane and Plasma Lipid Peroxidation

Lipid peroxidation levels were fluorometrically calculated as thiobarbituric acid-reactive substances (TBARS) in erythrocyte membrane and plasma according to Ohkawa et al. [32]. About 0.2 mL of erythrocyte membrane or plasma suspension was mixed with1.5 mL of 0.8% (*w*/*v*) 2-thiobarbituric acid, 1.5 mL of 20% (*v*/*v*) acetic acid solution (pH 3.5), 0.2 mL of 8.1% (*w*/*v*) sodium dodecyl sulfate, and 0.6 mL of deionized water. Then, vortex tubes were heated for 60 min in a boiling water bath. Following cooling, 5.0 mL of n-butanol : pyridine (15 : 1, *v*/*v*) and 1 mL of distilled water were added. Tubes were then shaken for 10 min and centrifuged for 10 min at 4,000 rpm. The organic upper layer was separated, and fluorescence was measured at 515 nm excitation and 553 nm emission (PerkinElmer LS-55 fluorescence spectrometer, Germany). For calibration, 1,3,3-tetraethoxypropane was used. Data derived from plasma and erythrocyte membrane TBARS levels are expressed as nanomole per milliliter of plasma and nanomole per milligram membrane protein, respectively.

### 2.9. Na^+^/K^+^-ATPase Assay

The total ATPase activity of erythrocyte was determined in its membrane. For this purpose, fifty microliters of erythrocyte membrane suspension was incubated for 90 min at 37°C in 25 mM KCl, 5 mM ATP, 75 mM NaCl, 0.1 mM EGTA, 5 mM MgCl2, and 25 mM Tris-HCl (pH 7.5) in a total volume of 500 *μ*L. Trichloroacetic acid was added to stop the reaction to a final concentration of 5% (*w*/*v*). An aliquot of the supernatant derived from centrifugation for 20 min at 1,500 g was used to estimate total liberated inorganic phosphorus from Fiske and Subbarow reaction [33]. This assay was repeated in the presence of an inhibitor of Na^+^/K^+^-ATPase activity (1 mM Ouabain). Total ATPase activity was reported as nmol of liberated inorganic phosphorus per milligram of membrane protein/hour. By subtracting the ATPase activity in the presence of ouabain from the total in the absence of ouabain, the activity of Na^+^/K^+^-ATPase was measured, too [30].

### 2.10. Measurement of Vitamins

An isocratic high-performance liquid chromatography (HPLC) method was used to determine serum level of vitamin A (retinol) and E (*α*-tocopherol) simultaneously using UV detection [34, 35]. Retinol acetate and tocopherol acetate were used as internal standards for retinol and *α*-tocopherol, respectively. The constituents of HPLC system consists are a LKB 2155 column oven, a LKB 2248 pump, and a 2140 rapid spectral detector (all of them are purchased from LKB, Pharmacia, Uppsala, Sweden). The isocratic mobile phase, methanol : acetonitrile : tetrahydrofuran (75 : 20 : 5), was pumped using a 5 *μ*m reverse phase column (Eurospher II 100-5 C18, 250 × 4.6 mm) (Knauer, Berlin, Germany) at a flow rate of 1.5 mL/min. *α*-tocopherol and retinol are monitored at 285 nm and 325 nm, respectively.

### 2.11. Data Analysis

Statistical analyses were performed using the SPSS version 26 software package (SPSS Inc., Chicago, IL, USA). Continuous variables are expressed as mean ± SD or as medians and interquartile ranges (IQR) in the case of skewed distributions. Categorical data are expressed as percentage. The Kolmogorov-Smirnov test was used to evaluate data regarding normal distribution. The intergroup differences regarding continuous variables were investigated using Mann–Whitney *U* test and independent *t* test. Chi-square test was used to compare categorical variables. The Pearson correlation test was used to evaluate correlations between variables. Significant difference was defined as *p* value less than 0.05.

## 3. Result

### 3.1. Subjects

Totally, 78 cases were enrolled in this study. Demographic and clinical characteristics of the participants are presented in Table 1. There were no significant differences regarding age, gender, and body mass index (BMI) between patients with OCD and age-matched healthy controls. A total of 39 patients (28 females, 11 males), with a mean age of 32.76 ± 12.32 years, were enrolled in this survey. The control group consisted of 33 women and 6 men (from 18 to 60 years and mean age of 27.25 ± 8.77 years). Four patients (10.3%) with rare OCD, 8 (20.5%) with mild OCD, 14 (35.9%) with moderate, and 13 (33.3%) with severe OCD types were diagnosed by Y-BOCS (Table 1).

### 3.2. MDA, Antioxidant, and Vitamin Levels

Our data showed a significant reduction in the levels of vitamin E (cases 49.27 ± 15.3, controls 57.23 ± 15.47, *p* = 0.024) and vitamin A (cases 4.82 ± 1.27, controls 6.15 ± 1.89, *p* = 0.001) in OCD patients compared with the control group. In addition, our findings showed a significant higher levels of MDA (serum: cases 4.68 ± 0.81, controls 3.96 ± 0.91, *p* < 0.0001/RBC membrane: cases 0.495 ± 0.079, controls 0.437 ± 0.057, *p* = 0.002) and had an insignificantly higher level of TAC (cases 910.42 ± 163.20, controls 865.14 ± 124.69, *p* = 0.190) and GSH (cases 36.82 ± 5.12, controls 35.70 ± 4.50, *p* = 0.306) compared to the controls.

### 3.3. ATPase Activity

The activity of erythrocyte membrane Na^+^/K^+^-ATPase was significantly diminished in OCD cases vs. control group (cases 373.57 ± 74.83, controls 441.32 ± 120.74, *p* < 0.0001), as summarized in Table 2.

### 3.4. Association between Parameters Related with Oxidative Stress

**Figure 1:**
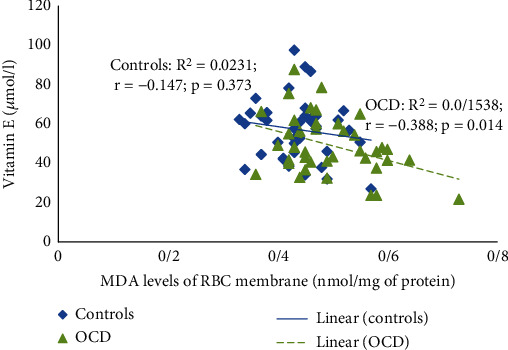
Correlations between vitamin E and MDA RBC membrane participants. MDA: malondialdehyde; OCD: obsessive-compulsive disorder; RBC: red blood cell.

Based on our data, we observed a significant negative relationship between RBC membrane MDA levels and vitamin E (*r* = –0.351, *p* = 0.002; Figure 1).

## 4. Discussion

We have investigated the association between clinical variables, levels of oxidative stress markers, and Na^+^/K^+^-ATPase activity in OCD patients. The main finding of this study revealed significant differences in the levels of oxidative stress markers between the two groups. Also, patients with OCD had lower ATPase activity compared to healthy controls. Some case control studies [36–43] and a meta-analysis [44] concentrated on the relationship between OCD and MDA. In contrast with our study, Ranjekar et al. [45] observed no significant differences in serum levels of lipid peroxidation markers between patients with schizophrenic and healthy control, and R. Kurup and P. Kurup [46] found that OCD patients had lower MDA level than controls. Also, Ersan et al. [37] and Shohag et al. [38] observed a significant decreased level of vitamin E in serum of OCD patients, but Shohag et al. did not observe any significant difference regarding serum vitamin A levels between OCD and healthy controls. A meta-analysis found an inverse association between vitamin E blood levels and OCD [47]. In the present study, serum GSH and TAC levels were not significantly different between the two groups. In line with our results, Behl et al. [36] found no significant difference in GSH levels between the OCD patients vs. controls. In contrast, Orhan et al. [40] showed a significantly lower GSH levels in OCD group compared to healthy individuals. OCD patients have been suggested to be exposed more often to premorbid environmental stressors, such as psychological stressors, perinatal events, and other traumatic events. These factors are thought to induce inflammation [48] and oxidative stress with potential induction of neurochemical modifications through various neurotransmitter pathways such as glutamate [49, 50].

Recently, it has been speculated that initially oxidative stress could be adaptive via enhanced neurotransmission, but it would exaggerate neurophysiological responses in long term with subsequent disruption of physiological neurotransmission and enhanced permeability of blood-brain barrier. These events would finally promote inflammatory neuronal damage and subsequent neuronal death [50, 51]. Thus, oxidative stress might function as a mediator between and disruption of cortico-striato-thalamo-cortical circuit and adverse life events in OCD. Mutually, oxidative stress might be a consequence of environmental exposures due to the OCD over behavior. Indeed, higher prevalence of anxiety disorders, metabolic syndrome, substance use disorders, mood disorders, and impulse control disorders has been observed in OCD patients [52, 53]. Besides, all of the above mentioned factors have a strong impact on lifestyle and are associated with induction and development of oxidative damage [54–56]. There are also some line of evidence supporting the reduced oxidant markers and enhanced antioxidant markers using antidepressants in the setting of OCD and major depression disorder [57, 58].

To the best of our knowledge, only one case-controlled study has investigated Na^+^/K^+^-ATPase activity in OCD subject [46]. In contrast with this study, we found that Na^+^/K^+^-ATPase activity was significantly lower in OCD patients than in controls. In line with our results, Banerjee et al. [59] found a significantly diminished activity of Na^+^/K^+^-ATPase in erythrocytes from patients with bipolar disorder. Kirshenbaum et al. [60] found that a mutation resulting in lower neuronal Na^+^/K^+^-ATPase activity would interact with stress exacerbating depression in mice. Furthermore, they observed a significant correlation between mood and Na^+^/K^+^-ATPase activity that could be related to both bipolar disorder and unipolar depression. Na^+^/K^+^-ATPase also function as a signal transducer besides its principal transport function. The involvement of Na^+^/K^+^-ATPase-mediated signaling has been reported in various physiological processes such as inflammation, cell growth, differentiation, kidney function, muscle contractility, and behavior. OCD can also be considered as a prototypical compulsivity disorder and kind of “behavioral addiction” [61, 62]. Numerous investigations have shown elicited behavioral alterations due to mutations in Na^+^/K^+^-ATPase isoform [24, 63–69]. Overall, data support the role of Na^+^/K^+^-ATPase activity in determining behavior.

This current study had several limitations. Firstly, oxidative markers could be influenced by various factors, such as age, smoking, or body mass index [70, 71]. We adjusted for several important confounding factors in our analysis, such as smoking and body mass index. Secondly, because the sampling of patients in the present study coincided with the outbreak of coronavirus, this may have an effect on our study, although all subjects had OCD before the onset of the pandemic. Thirdly, the patient group was interviewed by two independent psychiatrists, but the control group was just interviewed by a clinical psychologist, because they may have another psychiatric disorder (spatially personal disorder). Studies have shown that common tools for investigation of mental health are not accurate [72]. These cases seem to lead to the use of more reliable methods such as molecular methods to improve diagnosis (perhaps, it can help diagnose disorders at an early age). Indeed, none of the biomarkers have sufficient sensitivity and specificity as a diagnostic biomarker. Prediction of clinical outcomes seems to be a promising field of research pursuing appropriate biomarkers for OCD [73].

In conclusion, our findings indicate significantly higher levels of MDA in both serum and RBC membrane, lower levels of serum vitamins A and E, and lower activity of membrane Na^+^/K^+^-ATPase in OCD patients compared to controls. These findings suggest an imbalance between oxidant and antioxidant factors in OCD patients that might play a fundamental role in etiopathogenesis of OCD.

## Figures and Tables

**Table 1:** Demographic characteristics and clinical features of the participants.

Variable	Patients	Controls	*p* value
Number			
Overall	39	39	0.13
Men	11 (28.2%)	6 (15.4%)	
Women	28 (71.8%)	33 (84.6%)	
Age (years)	32.76 ± 12.32	27.25 ± 8.77	0.07
BMI (kg/m^2^)	25.8 ± 4.65	24.09 ± 4.74	0.11
Illness intuition	10.58 ± 10.35		
Duration of disease	7.27 ± 9.42		
Obsession	10.05 ± 4.43		
Compulsion	10.20 ± 3.95		
Y-BOCS			
Overall	20.25 ± 7.54		
Rare	4 (10.3%)		
Mild	8 (20.5%)		
Moderate	14 (35.9%)		
Severe	13 (33.3%)		
Status			
Single	19 (48.7%)	26 (66.7%)	0.14
Married	18 (46.2%)	13 (33.6%)	
Divorced	2 (5.1%)	0	
Race			
Pars	37 (94.9%)	36 (92.3%)	0.50
Other	2 (5.1%)	3 (7.7%)	

BMI: body mass index; Y-BOCS: Yale-Brown Obsessive-Compulsive Scale. Data are presented as percentages and mean ± SD. *p* value < 0.05 was considered as statistically significant.

**Table 2:** Level of oxidative stress markers and ATPase activity in the study population.

Marker	Patients	Controls	*p* value
Na^+^/K^+^-ATPase activity (nmol phosphorous liberated/mg protein/h)	373.57 ± 74.83	441.32 ± 120.74	<0.0001^∗^
MDA serum (nmol/L)	4.68 ± 0.81	3.96 ± 0.91	<0.0001^∗^
MDA RBC Membrane (nmol/mg of protein)	0.495 ± 0.079	0.437 ± 0.057	0.002^∗^
TAC (*μ*mol/L)	910.42 ± 163.20	865.14 ± 124.69	0.190
GSH (*μ*mol/L)	36.82 ± 5.12	35.70 ± 4.50	0.306
Vitamin A (*μ*mol/L)	4.82 ± 1.27	6.15 ± 1.89	0.001^∗^
Vitamin E (*μ*mol/L)	49.27 ± 15.3	57.23 ± 15.47	0.024^∗^
HDL (mg/dL)	44.02 ± 8.85	44.66 ± 7.85	0.736
LDL (mg/dL)	81 ± 23.47	74.82 ± 24.73	0.261
Cholesterol (mg/dL)	146.87 ± 34.12	137.38 ± 33.07	0.216
Triacylglycerol (mg/dL)	121.89 ± 83.15	89.33 ± 40.97	0.018^∗^

MDA: malondialdehyde; GSH: glutathione; TAC: total antioxidant capacity; LDL: low-density lipoprotein; HDL: high-density lipoprotein. Data are presented as mean ± SD. ^∗^*p* value < 0.05 was considered as statistically significant.

## Data Availability

Data and material will be provided upon request.
